# Function of m^5^C RNA methyltransferase NOP2 in high-grade serous ovarian cancer

**DOI:** 10.1080/15384047.2023.2263921

**Published:** 2023-10-06

**Authors:** Shimin Yang, Dongmei Zhou, Chunxiao Zhang, Jiangdong Xiang, Xiaowei Xi

**Affiliations:** Department of Gynecology and Obstetrics, Shanghai General Hospital, Shanghai Jiao Tong University School of Medicine, Shanghai, China

**Keywords:** m^5^C, NOP2, HGSOC, cAMP signaling pathway, RAPGEF4

## Abstract

RNA methyltransferase nucleolar protein p120 (NOP2), commonly referred to as NOP2/Sun RNA methyltransferase family member 1 (NSUN1), is involved in cell proliferation and is highly expressed in various cancers. However, its role in high-grade serous ovarian cancer (HGSOC) remains unclear. Our study investigated the expression of NOP2 in HGSOC tissues and normal fimbria tissues, and found that NOP2 was significantly upregulated in HGSOC tissues. Our experiments showed that NOP2 overexpression promoted cell proliferation *in vivo* and *in vitro* and increased the migration and invasion ability of HGSOC cells *in vitro*. Furthermore, we identified Rap guanine nucleotide exchange factor 4 (RAPGEF4) as a potential downstream target of NOP2 in HGSOC. Finally, our findings suggest that the regulation of NOP2 and RAPGEF4 may depend on m^5^C methylation levels.

## Introduction

Ovarian cancer is among the deadliest tumors in women, having the second highest incidence and highest mortality rate among female reproductive system tumors. It is also among the top five most lethal tumors worldwide.^1^ There are 239,000 new cases (3.6% of all cancer cases) and 152,000 deaths (4.3% of all cancer deaths) each year.^2^ The most common type of ovarian cancer is high-grade serous ovarian cancer (HGSOC), and it is now thought that most HGSOC originates in the fallopian tubes (FT).^3^ Unfortunately, the early symptoms of ovarian cancer are mild, which results in more than 60% of patients being found at an advanced stage. Standard treatments used to tackle ovarian cancer include cytoreductive surgery and chemotherapy based on carboplatin-paclitaxel combinations.^4^ Despite the advancements in surgery, chemotherapy, biochemical markers, genetic testing, and targeted therapies, almost all women suffer from ovarian cancer recurrences,^5^ and complications from other diseases are frequently a concern for advanced stage patients.^6^ As a result, researchers are exploring new mechanisms for early detection and improved diagnosis and therapy of HGSOC.

RNA methylation is a significant post-transcriptional modification of RNA. Current research focuses mainly on m^6^A, while research on m^5^C methylation is still in the early stages. RNA methylation plays a key role in various biological processes, such as nuclear RNA export capacity, RNA stability enhancement, RNA shearing and RNA-protein interaction regulation.^7^ Abnormal RNA methylation levels are associated with tumors,^8^ cardiovascular system diseases^9^ and neurological diseases.^10^ There are different types of RNA methylation, comprising N1-methyl-adenosine (m^1^A), m^5^C, N6-methyl-adenosine (m^6^A), 7-methyl-guanosine (m^7^G) and Um.^11^ m^5^C is methylation on the fifth C in the RNA cytosine. It is predominantly present in untranslated regions (3‘UTR and 5‘UTR), GC-enriched regions, adjacent to conserved AU(m^5^C)GANGUAGO sequences^12^ and near protein binding sites of AGO.^13^ RNA methylation is a reversible process whose regulation depends on writers (methyltransferases), erasers (demethylases), and readers (binding proteins). In m^5^C methylation, NOP2 is a vital methyltransferase that still lacks extensive research. NOP2 was initially explored as an important protein for ribosome synthesis and processing and 60s ribosomal subunit synthesis in budding yeast.^14^ Recently, NOP2 has been associated with ribosome biogenesis in humans^15^ and increased expression in most cancers resulting in a poor prognosis.^16^ NOP2 was found to have elevated expression in ovarian cancer compared to normal controls and was associated with poor prognosis.^17^ Moreover, NOP2 enhances the proliferation, migration and invasion in colon cancer.^18^ However, research on the mechanism of NOP2 in HGSOC has not yet been reported.

The cyclic adenosine monophosphate (cAMP) signaling pathway belongs to the cyclic nucleotide system, a signaling pathway that regulates the concentration of the second messenger through receptor binding to extracellular signals. The subsequent downstream responses include the induction of specific intracellular responses. cAMP is one of the signaling molecules that translates the extracellular signals into specific intracellular responses. Three primary targets of cAMP are protein kinase A (PKA), cAMP response element-binding protein (CREB), and exchange protein directly activated by cAMP (Epac). These processes involve gene transcription, cell migration ability, cell proliferation, cell death and play a crucial function in normal physiological activities and disease development.^19^ Recent studies have identified the cAMP signaling pathway as being closely related to tumorigenesis and progression and as a potential therapeutic strategy.^20^ Epac proteins are guanine nucleotide exchange factors (GEFs) for Rap1 and Rap2 and are associated with tumor development.^21–23^ Epac inhibitors are already available as a treatment to fight tumors.^24^

As a result of our study, we found that NOP2 was overexpressed in HGSOC compared to FT tissues. Furthermore, NOP2 could promote the migration and invasion of HGSOC cells *in vitro*, as well as the proliferation ability of cells *in vivo* and *in vitro*. Our experiments reveal that NOP2 regulates Rap guanine nucleotide exchange factor 4 (RAPGEF4) in HGSOC as a potential downstream gene. We further discovered that NOP2-induced proliferation of HGSOC cells was contingent upon the expression of RAPGEF4, which in turn was regulated by m^5^C methylation. Our findings indicate that a regulatory mechanism of NOP2 on RAPGEF4 could account for HGSOC carcinogenesis and progression via a novel mechanism.

## Materials and Methods

### Data resources

Matrix files in SOFT format for Gene Expression Omnibus (GEO) dataset (GSE10971)^25^ was downloaded from Gene Expression Omnibus (https://www.ncbi.nlm.nih.gov/geo/). Ovarian cancer cell line data was downloaded from Expression Atlas (https://www.ebi.ac.uk/gxa/home)^26^ and the accession number is PXD030304.^27^ The m^5^C regulator-associated somatic mutation and Copy number variation (CNV) analyses of pan-cancer and ovarian cancer (OV) were performed through the cBioPortal website (www.cbioportal.org).^28^ This data analysis included 398 TCGA ovarian cancer samples. Kaplan – Meier overall survival (OS) analysis of NOP2 expression in OV patients was performed through Kaplan-Meier Plotter (https://kmplot.com/analysis/).^29^

### Analysis of differentially expressed genes

The differential analysis of genes was implemented by the limma R package (version 3.52.4).^30^ The package is also implemented to calculate the up-regulated differentially expressed genes (DEGs) in different clusters. DEGs were determined for genes with *p* < .05 & |log_2_FC|≥1.

### Clinical sample

Between 2017 and 2022, 63 HGSOC tissues and 13 FT tissues were collected through Shanghai First People’s Hospital. All operations were performed after informing the patients and obtaining their consent. Ethical approval was obtained from the Medical Ethics Committee of Shanghai First People’s Hospital (ethics number: 2017KY068).

Table 1 Characteristics of the HGSOC patients.

**Table 1.** Characteristics of the HGSOC patients.

Characteristic	Levels	Overall
n		63
Tumor size (cm), n (%)	<3 cm	13 (20.6%)
	≥3 cm	50 (79.4%)
Age, n (%)	<50	8 (12.7%)
	≥50	55 (87.3%)
FIGO staging, n (%)	I	1 (1.6%)
	II	7 (11.1%)
	III	51 (81.0%)
	IV	4 (6.3%)
CA125 (U/ml), n (%)	<30	3 (4.8%)
	≥30	60 (95.2%)
HE4 (pmol/L), n (%)	<140	18 (28.6%)
	≥140	45 (71.4%)

### Wax block embedding and production of tissue microarrays

We fixed the collected tissue specimens in tissue fixative for more than 24 h. We removed the tissues from the fixative in a fume hood and trimmed the tissues with a scalpel, placing the prepared tissues and corresponding labels in an embedding box. The tissues were dehydrated by sequential immersion in graded concentrations of ethanol and xylene. All dehydrated tissues were submerged in pre-heated liquefied wax and immersed overnight. Tissue wax blocks were subsequently made on a HistoCore Arcadia (HistoCore Arcadia, Leica, Germany). Finally, we submitted the prepared tissue wax blocks to Servicebio (Wuhan, China) for tissue microarrays.

### Tissue wax sectioning

We cooled the prepared wax blocks on ice, and then placed the cooled blocks in a paraffin slicer (FINESSE 325, Thermo Scientific, USA) for slicing (4 μm thickness). Finally, we float the sections on warm water at 40°C to flatten the tissue, lift the tissue with a slide and bake the sections at 60°C. Sections can be stored at 4°C after cooling down naturally.

### Immunohistochemistry (IHC)

The previously prepared sections or tissue microarrays were placed in a Thermostatic Incubator (DNP-9052, JINGHONG, Shanghai) at 60°C for 3 hours or overnight to melt off the surface sealing wax. Sections or tissue microarrays were sequentially deparaffinized and rehydrated by immersion in xylene and a concentration gradient of ethanol in a fume hood. Citrate Antigen Retrieval Solution (P0081; Beyotime, China) was used for antigen retrieval in boiling water for 7 min. 3% hydrogen peroxide solution was used to eliminate endogenous peroxidase activity in tissues. Tissues were blocked with 10% goat serum (C0265; Beyotime, China) for an hour. Then, the anti-NOP2 antibody (10448–1-AP, Proteintech, China) diluted to 1:300 was added for overnight incubation at 4°C after PBS rinsing. After the tissue sections were returned to room temperature and rinsed with PBS, we used the immunohistochemistry kit (GK500705; Gene Tech, China) for secondary antibody incubation and staining. Then, tissue sections were stained again in hematoxylin (C0107; Beyotime, China) for 3 min and terminated staining in distilled water. Tissue sections were dehydrated by sequential immersion in a gradient of ethanol and xylene. Finally, the slices were blocked with neutral resin (GT100519; Gene Tech, China). Figures were captured with a fluorescent microscope (DM2500, Leica, UK). The results were analyzed and calculated the average optical density (AOD) values by Image-Pro Plus (version 6.0; Media Cybernetics, Rockville, Md). We open the picture in the software, click measure → intensity, click new → std. optical density → options → image in the intensity box, and then select the blank place in the figure, click the ok button. We change the incidental level to the value of blank place and then click measure→count/size→select colors, click the icon of the pen on the left side, select the immunohistochemistry picture of the positive protein expression region, and click close after the selection. Finally get the mean of area and mean of integrated optical density (IOD) by clicking Measure→Select Measurements, selecting iod→ok→count inside and clicking view→statistic in the table. AOD=mean of IOD/mean of area.

### Cell growth and cell culture

Hey (RRID: CVCL_0297), Caov3 (RRID: CVCL_0201), 293T (RRID: CVCL_0063) and Tubal Epithelial Cells (RRID: CVCL_F597) were provided by National Collection of Authenticated Cell Cultures (Shanghai, China). Hey, Caov3, 293T and Tubal Epithelial Cells were cultured using high sugar DMEM medium (319–005-CL; MUTICELL, China) containing 10% fetal bovine serum (086–150; MUTICELL, China). The growth environment was 37°C and 5% CO2 in a CO2 - Incubator (51023126, Thermo Fisher Scientific, USA).

### Plasmid construction and transfection

The plasmid of NOP2 knockdown was synthesized by Tsingke Biotechnology (Beijing, China) and the plasmid has been validated by DNA sequencing. The plasmid of NOP2 and RAPGEF4 overexpression was synthesized by Genomeditech (Shanghai, China) and the plasmid has been validated by DNA sequencing. DNA sequencing report in Supplementary Material. Briefly, the shRNA primer pair for NOP2 was derived from shRNAlibrary (TRC) and inserted into pLKO.1 vector to generate the shNOP2 plasmid. The open reading frame of NOP2 was copied and inserted into PGMLV vector to generate the NOP2 overexpression plasmid. The open reading frame of RAPGEF4 was copied and inserted into the pcDNA3.1 vector to generate the NOP2 overexpression plasmid. Cells transfected with the NOP2 knockdown plasmid are referred to as shNOP2–1 and shNOP2–2, and those transfected with the control plasmid are referred to as shNC. Cells transfected with the NOP2 overexpression plasmid are referred to as NOP2, and those transfected with the control plasmid are referred to as NC. Cells transfected with both the NOP2 knockdown plasmid and RAPGEF4 overexpression plasmid are referred to as shNOP2- RAPGEF4, and those transfected with the control plasmid are called shNOP2-NC.

293T was cultured in 6-cm dishes in advance to a cell density of 50%. Transfection was performed using lipofectamine 3000 reagent (Invitrogen Life Technologies®, USA) according to the manufacturer’s protocol. 293T was incubated at 37°C, 5% CO2 for 48 h. Subsequently, the supernatant was collected and filtered after centrifugation at 1,000×g for 3 min by centrifuge (Allegra X-30 R, BECKMAN COULTER, USA) to obtain the lentiviral solution. The lentiviral solution was diluted in different concentration gradients (200ul, 400ul,800ul) and added into six-well plates of Hey and Caov3 cells that were cultured to 40% cell density in advance, respectively. The cells were cultured at 37°C, 5% CO2 for 48 h and subsequently replaced with 4 ml of DMEM containing puromycin (2 mg/ml) for screening. After 24-48 h of incubation at 37°C with 5% CO2, the most surviving cells in the six-well plate were selected for expansion. Subsequent validation was performed by Western Blot and qPCR assays. The sequence of short hairpin ribonucleic acid (shRNA) is as follows:

shNOP2–1: 5′- GACGATGCTGATACGGTAGAT −3′

shNOP2–2: 5′- CACTGTACCTTCTGTCACAAA −3′

### Western blot and antibody

Cells were incubated in RIPA lysis buffer (Beyotime, China) containing 1% phenylmethanesulfonyl fluoride (PMSF) and 0.1% protease inhibitor cocktail (Beyotime, China) at 4°C for 30 min, centrifuged at 4°C for 15 min at 13,000×g. Protein concentration was determined using BCA Protein Assay Kit (Beyotime, China) according to the manufacturer’s protocol. Finally, SDS-PAGE Protein Loading Buffer (5X) (Beyotime, China) was added at a proportion of 4:1 and incubated at 95°C for 10 min.

PAGE Gel Fast Preparation Kit (Epizyme, China) was used to prepare a 10% gel. 25 ug of total protein per well was sampled and transferred to methanol-activated polyvinylidene fluoride membranes (PVDF; Millipore, USA) after electrophoresis. After the end of the transfer, the membrane was put into 5% nonfat milk (E504BA0014, BBI Life Sciences, China), blocked at room temperature for 1 h, and then rinsed three times with 0.1% Tris-HCl plus Tween-20 (TBST) for 5 min each. The membranes were incubated with primary antibody and secondary antibody, and rinsed three times after each step. The membranes were tested using the ECL luminol kit (BioVision, USA) by Chemiluminescent imaging system (Tanon 5200; Tanon, China). The antibodies and dilutions used in the experiment are as follows:

Anti-NOP2 antibody (10448–1-AP) diluted to 1:1000, anti-GAPDH antibody (60004–1-lg) diluted to 1:50000, Anti-rabbit (SA00001–2) diluted to 1:2000 and anti-mouse (SA00001–1) diluted to 1:2000 secondary antibodies were provided by Proteintech (China); Anti-RAPGEF4 antibody (HPA028968-25UL) diluted to 1:1000 was provided by Sigma-Aldrich (USA). Relative protein levels were compared quantitatively with GAPDH using ImageJ software (version 1.52a; National Institutes of Health).^31^

### RNA extraction and qPCR

Total RNA was extracted from Hey and Caov3 cell lines using TRIeasy™ LS Total RNA Extraction Reagent (19201ES60; Yeasen, China) based on the manufacturer’s protocol. RNA was reverse transcribed into cDNA using reverse transcription reagent (R202–02; EnzyArtisan, China). NOP2, RAPGEF4 and GAPDH were detected by qRT-PCR amplification using Universal SYBR qPCR Mix (Q204; EnzyArtisan, China) under QuantStudio 6 and 7 Flex and ViiA 7 Real-Time PCR Systems (Thermo Fisher Scientific, USA). The qPCR cycling conditions were as follows:

1. Pre-denaturation: 95°C, 30s, cycle number: 1
2. Denaturation: 95°C, 10s; annealing & extension: 60°C, 30s, cycle number: 40
3. Melting curve stage: step 1: 95°C, 15s; step 2: 60°C, 60s; step 3: 95°C, 30s mRNA expression was normalized and calculated using the 2-ΔΔCt method^32^. Primers’ sequences used were as follows:

NOP2, Forward primer: 5’-TGTCTGAGCTGGTGGAGTTCTTAG-3’

Reverse primer:5’-ACCCCACGATTGATTAGAGCC-3’

GAPDH, Forward primer:5’-CAGGGCTGCTTTTAACTCTGGTAA-3’

Reverse primer:5’- GGGTGGAATCATATTGGAACATGT-3’

MeRIP-RAPGEF4, Forward primer:5’-TTTTGTTCGTATTGTTTTTTATTGC-3’

Reverseprimer:5’-CATAAATCTTCCTCAACAACTATCGA-3’

### RNA m^5^C dot blotting assay

RNA was extracted from NOP2 overexpression and knockdown cells and the corresponding control cells and its mass was calculated. Then the RNA secondary structure was disrupted by incubation at 95°C for 5 min. Setting different concentration titers, RNA was added to the nylon membrane (20G00109; Merck, Germany) by 2ul drops per well. After cross-linking at 254 nm UV for 30 minutes and thermal cross-linking in an oven at 60°C for 1 h, the membrane was blocked with 5% nonfat milk. This was followed by incubation with anti-m^5^C primary antibody (68301–1-lg, Proteintech. China) diluted to 1:2500 and the anti-mouse secondary antibody diluted to 1:2000. Finally, the membranes were assayed using the ECL luminol kit (K824; BioVision, USA) by chemiluminescent imaging system (Tanon 5200; Tanon, China).

### Cell proliferation assay

According to the manufacturer’s protocol, cell proliferation assays were assessed using Cell Counting Kit-8 (C0038; Beyotime, China). The cells were added to 96-well plates at 2000 cells per well and then processed at 0,24,48, and 72 hours. CCK-8 reagent was added to the cells and incubated in the incubator for 1 h. Measuring the optical density (OD) at 450 nm using the Varioskan LUX multimode microplate reader (Thermo Fisher Scientific, USA). DMEM containing 10% FBS was used as a blank control.

### Colony-formation assay

A total of 800 transfected Hey and 1500 Caov3 cells were seeded in six well-plates during their logarithmic growth phase. They were incubated in DMEM medium containing 10% FBS at 37°C with 5% CO2 until 14 days or the vast majority of colonies with cell numbers > 40. The cells were then fixed with 4% paraformaldehyde (BL539A; Biosharp, China) for 30 min and stained with crystal violet staining solution (C0121; Beyotime, China) for 20 min. Figures were captured with a camera (M6, Canon, Japan)

### Transwell cell migration assay

60 μl of Matrigel matrix (356234; Corning, USA) diluted to 1:6 with serum-free medium was added to the upper transwell chambers (14421030; Corning, USA) and placed in 37°C for 1 hour to allow it to solidify before use. 200 μl of serum-free medium containing 1 × 10^5^ Hey cells and 200 μl of serum-free medium containing 1 × 10^5^ Caov3 cells were added to the upper chambers, and 700 μl of DMEM medium containing 10% FBS was added to the lower layer of the chambers. Hey cells cultured for 24 hours (Caov3 cells at 48 hours) were subjected to migration assay and cultured for 48 hours (Caov3 cells at 72 hours) were subjected to invasion assay. Finally, the cells were then fixed with 4% paraformaldehyde for 30 min and stained with crystal violet staining solution for 20 min. Figures were captured with a fluorescent microscope (DM2500, Leica, UK)

### Tumor xenograft

BALB/c female nude mice, 4–6 weeks old, were purchased from the Experimental Animal Center of Shanghai First People’s Hospital and randomly divided into two groups (seven mice in each group) for the experiments. Stable NOP2 knockdown Hey cells and the control cells (1 × 10^7^ cells, 100 μl PBS) were injected into the axillary region of BALB/c female nude mice. After tumor formation, tumor volume (1/2 × length × width^2^) was measured every 2 days. Tumor tissue was collected after 3 weeks and tumor volume and weight were measured. Tumor tissues were embedded, sectioned and stained as described above. All procedures were approved by the Experimental Animal Center of Shanghai First People’s Hospital (ethics number: 2022AW028).

### RNA-seq

RNA was extracted from stable NOP2 knockdown Hey cells and the control cells using TRIzol (15596018; thermofisher, USA) according to the manufacturer’s protocol. RNA-seq performed by LC Sciences (Hangzhou, China). The detailed experimental procedure is as follows: The amount and purity of total RNA was controlled with a NanoDrop ND-1000 (NanoDrop; Wilmington, DE, USA) and the integrity of RNA was examined by a Bioanalyzer 2100 (Agilent, CA, USA). Concentrations > 50ng/μl, the value of RIN > 7.0, and total RNA >1 μg were sufficient for downstream assays. The polyadenylated (PolyA) mRNA was specifically captured by two rounds of purification using oligo (dT) magnetic beads (cat.25–61005; Thermo Fisher, USA). Captured mRNA was fragmented using the Magnesium Ion Interruption Kit (NEBNext^R^ Magnesium RNA Fragmentation Module, cat. E6150S; USA) at high temperature, 94°C for 5–7 min. The fragmented RNA was incubated with reverse transcriptase (Invitrogen SuperScriptTM II Reverse Transcriptase, cat.1896649, CA, USA) to synthesize the cDNA. These complex double strands of DNA and RNA were then converted into DNA duplexes using E. coli DNA polymerase I (NEB, cat.m0209, USA) with RNase H (NEB, cat.m0297, USA) for two-strand synthesis. At the same time, dUTP Solution (Thermo Fisher, cat.R0133, CA, USA) was spiked into the second strand to complement the ends of the double-stranded DNA to flat ends. A base is then added to each end to enable it to be ligated to a junction with a T base at the end, and the fragment size is screened and purified using magnetic beads. The second strand was digested with UDG enzyme (NEB, cat.m0280, MA, US) and then denatured by PCR-pre-denaturation at 95°C for 3 min, 98°C for a total of 8 cycles of 15 s each, annealing to 60°C for 15 s, extending at 72°C for 30 s, and a final extension retained at 72°C for 5 min to form a fragment size of 300 bp ±50bp (strand-specific library). Finally, it was bipartite sequenced using illumina NovaseqTM 6000 (LC Bio Technology CO., Ltd. Hangzhou, China) according to the standard operation, and the sequencing mode was PE150. The final data obtained were filtered to obtain high-quality sequencing data (Clean Data) by Cutadapt (version 1.9; https://cutadapt.readthedocs.io/en/stable/).^33^ The parameters were as follows:1. removing reads containing adapters; 2. removing reads containing polyA and polyG; 3. removing reads containing more than 5% of unknown nucleotides (N); 4. removing low quality reads containing more than 20% of low quality (Q-value ≤20) bases. Then sequence quality was verified using FastQC (version 0.11.9; http://www.bioinformatics.babraham.ac.uk/projects/fastqc/) including the Q20, Q30 and GC-content of the clean data. We compared the Clean Data to the human reference genome using the HISAT2 (version 2.2.1) R package.^34^ Three biological replicates were included for each sample in this experiment. Functional analysis of DEGs was performed by Gene Ontology (GO) and Kyoto Encyclopedia of Genes and Genomes (KEGG) using the ClusterProfiler (version 4.4.4) R package,^35^ and gene set enrichment analysis (GSEA) was performed using the GSEABase (version 1.58.0; https://www.bioconductor.org/packages/release/bioc/html/GSEABase.html) R package.^36^ The threshold for screening DEGs is as follows: |log2(Foldchange)| > 1, q-value <0.05.

### Extraction of cytoplasmic and nuclear lysates

Nuclear and cytoplasmic proteins were extracted by Nuclear and Cytoplasmic Protein Extraction Kit (P0027, Beyotime, China) to assess the distribution of NOP2 in the cells. In brief, the apposed cells were treated with EDTA solution and collected by pipetting. Cytoplasmic proteins were obtained by adding cytoplasmic protein extraction reagent, incubating on ice for 15 min, and centrifuging at 16,000×g for 5 min. Then the nuclear extraction reagent was added and after 30 min of intermittent vigorous vortex, the nuclear proteins were obtained by centrifuging at 16,000×g for 10 min. The final obtained nuclear and cytoplasmic proteins were verified by Western Blot assay. Anti-LaminB1 antibody (66095–1-lg; Proteintech, China) diluted to 1:5000 was used for the nuclear proteins.

### m^5^C RNA RIP assay

The GenSeq@m^5^C MeRIP kit (GS-ET-003; Cloud-seq, China) was used to perform specific enrichment of m^5^C-modified regions in the transcriptome according to the manufacturer’s protocol. Briefly, RNA was first extracted from cells using the method proposed above. RNA was fragmented (RNA fragment size ~ 200 nucleotide), and the fragmented RNA was incubated with pre-prepared immunoprecipitated magnetic beads for 1 h. The precipitated RNA was eluted from the magnetic beads and purified. Finally, the measurement of m^5^C methylation enriched fragment RNA and input levels by qRT-PCR. MeRIP-qPCR-related primers for RAPGEF4 were obtained by screening through the UCSC genome browser website (http://genome.ucsc.edu/cgi-bin/hgGateway)^37^ and Meth Primer website (http://www.urogene.org/methprimer/).^38^

### Co-immunoprecipitation (CO-IP)

The CO-IP assay was completed using the Protein A+G Magnetic Bead Immunoprecipitation Kit (P2179S; Beyotime, China) according to the manufacturer’s protocol. Briefly, the cells were lysed with a lysate containing inhibitors. The pre-prepared beads were incubated with the antibody for 1 h at room temperature. The samples were then incubated overnight at 4°C with the antibody-conjugated magnetic beads. Finally, the precipitated protein samples were eluted from the magnetic beads. The antibody used in CO-IP assay is Anti-NOP2 antibody (ab271075; Abcam, UK) diluted to 1:30. The final result was detected by Western Blot assay.

### Statistics

All experiments in this study were repeated three times. Any special cases will be described separately. All of our data were first analyzed for normal distribution by performing the Shapiro-Wilk test. The unpaired and two-tailed student’s t-tests was used for normally distributed data. The Mann-Whitne test was used for non-normally distributed data. All statistical analyses were conducted with SPSS software (version 19.0; Chicago, IL, USA). Analysis of RNA-seq data and GEO data was performed by the R (version 4.0.3; https://www.r-project.org/). P < .05 was regarded as statistically significant (*P <.05, **P <.01, ***P <.001). All the graphs were produced using GraphPad Prism software (version 8.0; San Diego, CA).

## Results

### NOP2 was upregulated in human HGSOC compared to FT

**Figure 1.**
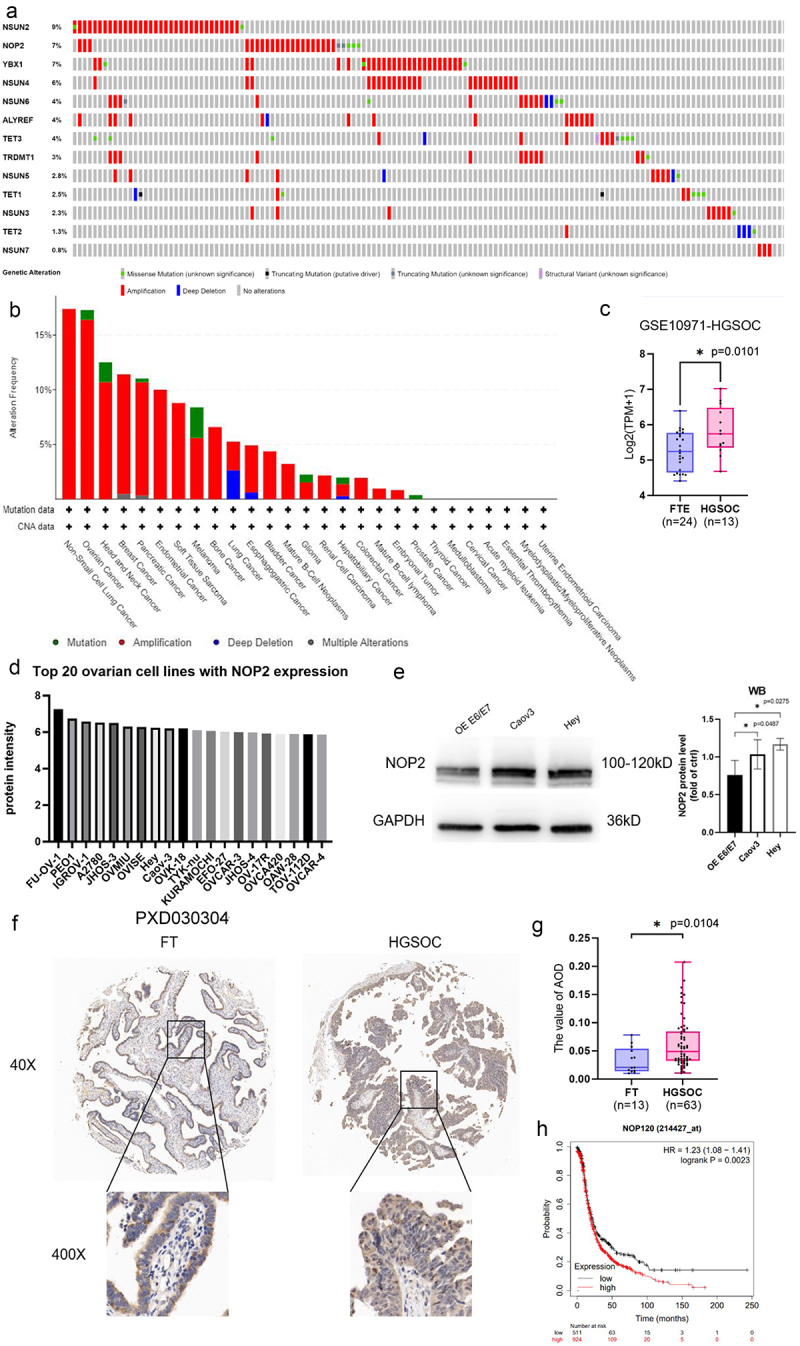
NOP2 and RNA m^5^C-related regulators expression status in HGSOC. (a) mutation, structural variant and copy number variation in m^5^C regulators in ovarian cancer. The figure was downloaded from the cBioportal website (www.cbioportal.org). This analysis contains 398 samples, and only the samples with mutation and copy number variation are shown in the figure. The rest of the unshown parts are no alterations. (b) mutation, structural variant and copy number variation in NOP2 in pan-cancer. The figure was downloaded from the cBioportal website (www.cbioportal.org). (c) differential expression analysis of NOP2 in HGSOC and FTE in GEO dataset GSE10971. (d) NOP2 protein expression in the top 20 ovarian cancer cell lines in expression Atlas dataset PXD030304. (e) Western blot analysis of NOP2 expression in Hey, Caov3 and Tubal Epithelial cells OE E6/E7. (f) Representative images of IHC staining of NOP2 expression levels in tissue microarray (×40: scale bar = 100 μm; ×400: scale bar = 10 μm). (g) differential expression of NOP2 between HGSOC and FT. (h) Kaplan – Meier OS analysis of NOP2 expression in OV patients from Kaplan-Meier Plotter. *: *p* < .05.

Firstly, mutations and amplifications in OV were dominated by NSUN2, NOP2, and YBX1, with percentages of 9%, 7%, and 7%, respectively (Figure 1a). In the pan-cancer data on genes coding for RNA m^5^C regulatory proteins, it was found that the mutations and amplifications of NOP2 in ovarian cancer were the second highest (Figure 1b). Additionally, analysis of GEO dataset GSE10971 revealed that NOP2 expression levels were markedly elevated in HGSOC in comparison to fallopian tube epithelium (FTE) (Figure 1c). We analyzed the expression of NOP2 protein in ovarian cancer cell lines through the Expression Atlas dataset P×D030304and found that the HGSOC cell lines Hey and Caov3 were ranked eighth and ninth, respectively (Figure 1d). We found that NOP2 expression was elevated in Hey and Caov3 cells compared to human fallopian tube cells OE E6/E7 by Western Blot assay (Figure 1e). Therefore, we chose Hey and Caov3 cell lines to complete the subsequent experiments. To further investigate the expression level and localization of NOP2 in HGSOC, we performed IHC staining of our HGSOC tissue microarray (Supplementary Figure S1 C). The results showed that NOP2 was expressed in both nucleus and cytoplasm, mainly distributed in the nucleus (Figure 1f). Furthermore, NOP2 was upregulated in HGSOC tissues compared to FT tissues (Figure 1g). The Kaplan-Meier overall survival analysis revealed that patients in the high-NOP2 expression group had significantly poorer overall survival than those in the low-NOP2 expression group (Figure 1h).

### *NOP2 enhances the proliferation, migration and invasive ability of HGSOC cells* in vitro

**Figure 2.**
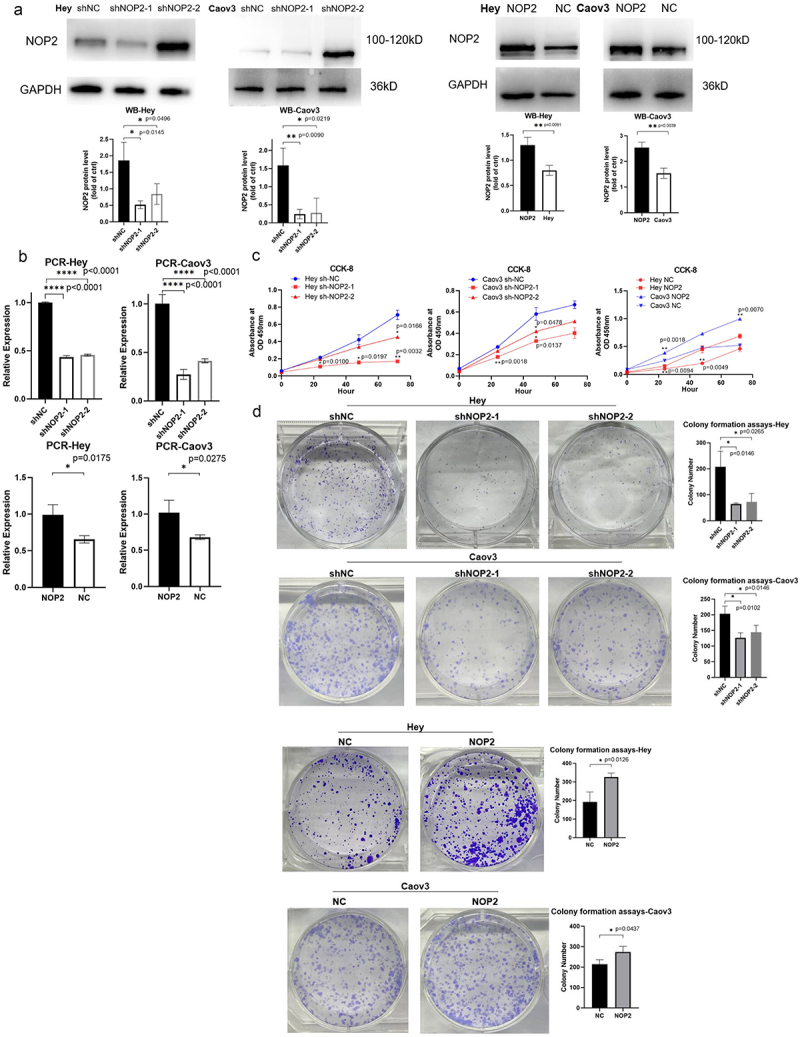
NOP2 enhances the proliferation ability of HGSOC cells *in vitro*. (a) Western blot analysis of stable NOP2 expression knockdown and overexpression in Hey and Caov3 cells. (b) qRT-PCR analysis of stable NOP2 knockdown and overexpression Hey and Caov3 cells. (c) CCK-8 cell proliferation assay was conducted to determine the relationship between NOP2 expression and growth ability. (d) colony formation assay was conducted to ascertain the relationship between NOP2 expression and clone formation ability. *: *p* < .05, **: *p* < .01, ****: *p* < .0001.

First, we performed Western blot and qRT-PCR assays to confirm stable knockdown or overexpression of NOP2 in Hey and Caov3 cells Figure 2(a,b). Subsequently, we conducted a CCK-8 assay to assess cell proliferation, which revealed that NOP2 knockdown significantly inhibited cell proliferation, while NOP2 overexpression promoted cell proliferation (Figure 2c). In addition, colony formation assays showed that colony formation was greatly inhibited by reduced expression levels of NOP2, and colony formation was promoted by increased expression levels of NOP2 (Figure 2d).

**Figure 3.**
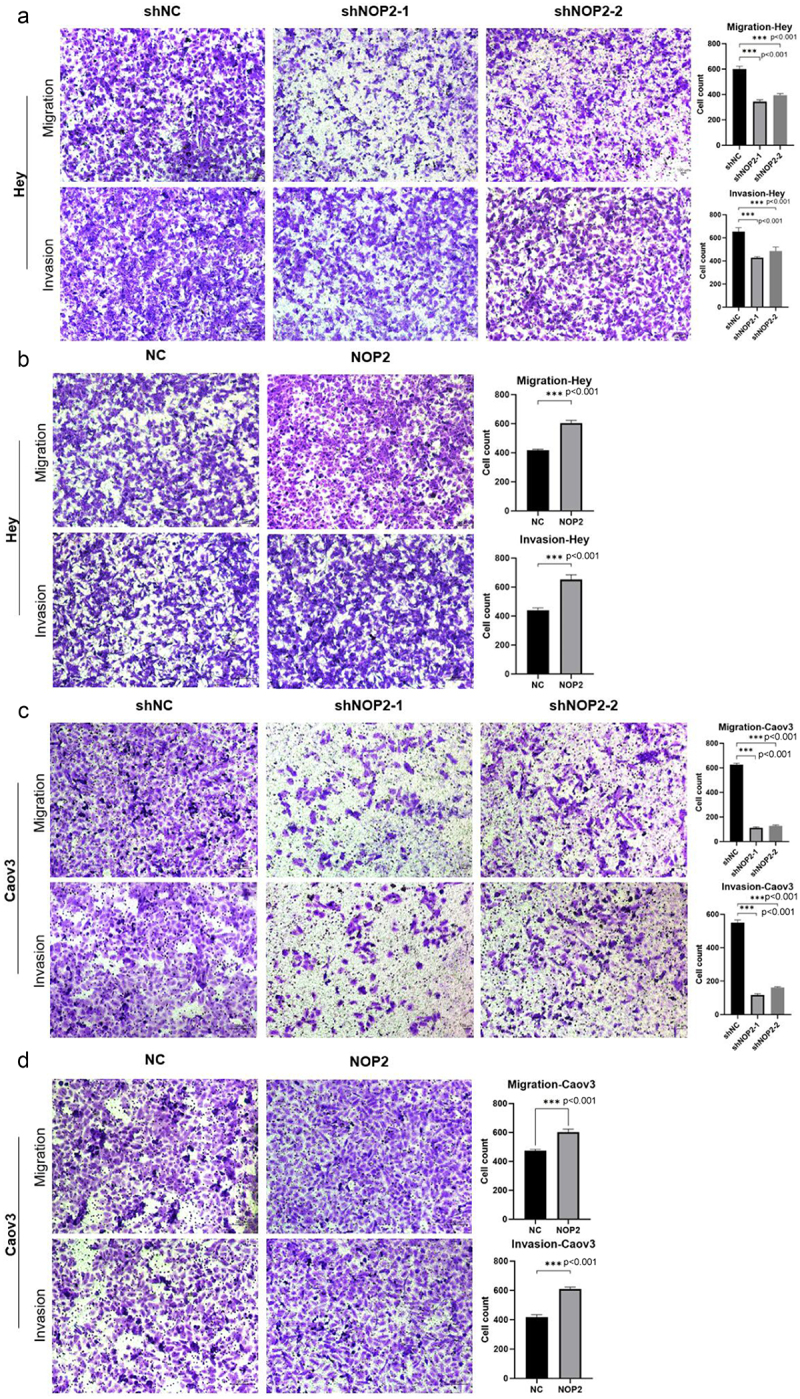
NOP2 enhances the migration and invasive ability of HGSOC cells *in vitro*. (a-b) cell migration assay and cell invasion assay was performed to determine the migration and invasion capacity of NOP2 knockdown and overexpression in Hey. (c-d) cell migration assay and cell invasion assay was performed to determine the migration and invasion capacity of NOP2 knockdown and overexpression in Caov3. ***: *p* < .001.

Additionally, the cell migration assay showed that NOP2 knockdown significantly inhibited cell migration, while NOP2 overexpression promoted cell migration. Furthermore, cell invasion assays showed that decreased NOP2 expression obviously inhibited cell invasion, while increased NOP2 expression promoted cell invasion (Figure 3a-d). Our results suggest that NOP2 plays an essential role in the proliferation, migration and invasion ability of HGSOC cells *in vitro*.

### *NOP2 promotes tumorigenesis in HGSOC cells* in vivo

**Figure 4.**
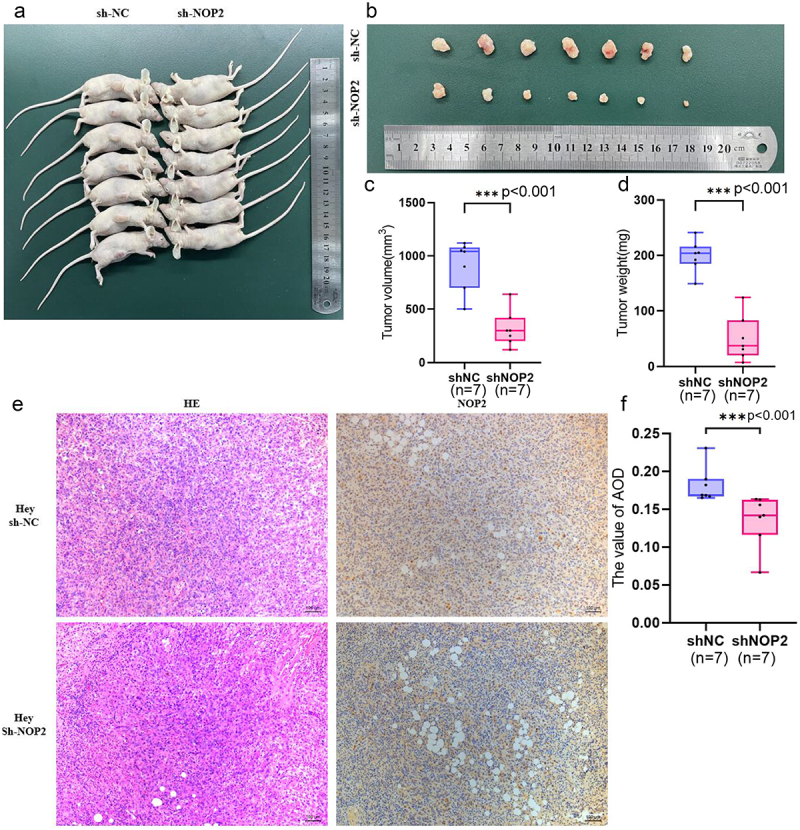
NOP2 promotes ovarian cancer oncogenesis *in vivo*. (a) stable NOP2 expression knockdown Hey cells and control cells were injected subcutaneously into BALB/c female nude mice. (b) BALB/c female nude mice carrying tumors. (c-d) the weight and size of tumors were measured after 3 weeks. (e) HE and IHC staining of NOP2 expression in tumor slices under × 200 magnification. (f) differential expression of NOP2 between shNOP2 tumors and shNOP2 tumors. Data were presented as the mean ± SD; *: *p* < .05. ***: *p* < .001.

In vivo experiment, stable NOP2 knockdown Hey cells and their control cells were injected subcutaneously into the axillae of BALB/c female nude mice. We observed that NOP2 expression reduction visually inhibited the growth of subcutaneous tumors in BALB/c female nude mice within three weeks Figure 4(a,b). After tumor excision, the mean tumor weight and volume were significantly lower in the knockdown group compared to the control group Figure 4(c,d). Moreover, tumors in the control group exhibited stronger NOP2 staining than the NOP2 knockdown cell group, as confirmed by IHC analysis Figure 4(e,f). Our findings suggest that NOP2 can promote HGSOC tumorigenesis *in vivo*.

### Potential targets of RAPGEF4 identified by RNA-seq as regulated by NOP2

To search for potential downstream targets of NOP2, we performed RNA sequencing to examine changes in mRNA expression in stable NOP2 knockdown Hey cells and their control cells. KEGG analysis of differential genes in our sequencing results showed that NOP2 may play a significant role in the cAMP second messenger-related pathway (Table 2) (Supplementary Figure S2 A-B). Given the multifaceted role of cAMP in the cell, we selected the cAMP signaling pathway as the target of NOP2 for further investigation. Our analysis suggests that the expression of RAPGEF4 in the cAMP signaling pathway is obviously decreased in ovarian cancer cells with stable knockdown of NOP2 expression compared to control cells (Table 3). Thus, we identified RAPGEF4 as a potential target regulated by NOP2.

**Table 2.** The results of KEGG analysis of differential genes were tabulated, indicating that the cAMP signaling pathway was significantly associated with NOP2.

Pathway	Input number	Background number	p-value
Leukocyte transendothelial migration	98	118	.008
Inflammatory bowel disease	46	145	.012
Phospholipase D signaling pathway	127	159	.015
Th1 and Th2 cell differentiation	73	168	.016
Osteoclast differentiation	107	187	.020
Rheumatoid arthritis	71	189	.021
Phenylalanine metabolism	12	18	.021
Rap1 signaling pathway	187	211	.025
cAMP signaling pathway	167	**222**	.028
Histidine metabolism	18	24	.028
beta-Alanine metabolism	27	31	.037
Tuberculosis	143	260	.037
Cell adhesion molecules	111	263	.038
Tyrosine metabolism	27	39	.046

The bolded row in the table is to emphasize the direction of the study.

**Table 3.** Differential genes enriched in cAMP signaling pathway of RNA-Seq.

Gene name	log_2_(FC)	p-value	Regulation
**RAPGEF4**	**−8.52**	＜.01	**down**
GRIA1	6.77	.04	up
ADORA2A	−4.01	＜.01	down
EDNRA	0.54	＜.01	up
SOX9	0.48	.01	up
PPARA	0.39	.02	up
PIK3R2	0.37	＜.01	up
ATP2B4	0.34	.01	up
EP300	0.33	.02	up
CREB3L2	0.29	.01	up
AFDN	0.28	.03	up
PIK3CB	0.28	.01	up
RRAS2	0.28	＜.01	up
GLI3	0.27	.04	up
EDN1	−0.23	.03	down

The bolded row in the table is to emphasize the direction of the study.

### A regulatory mechanism of NOP2 on RAPGEF4 that is dependent on the m^5^C methylation level

**Figure 5.**
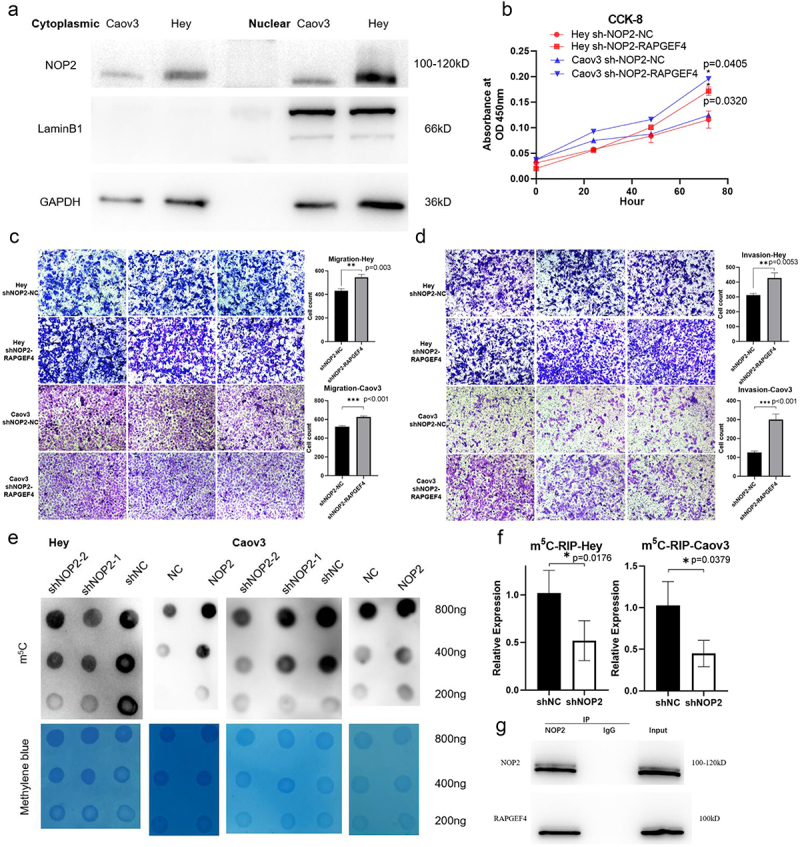
A regulatory mechanism of NOP2 on RAPGEF4 that is dependent on the m^5^C methylation level. (a) Extraction of cytoplasmic and nuclear lysates and Western Blot assay to detect NOP2 protein distribution in Hey and Caov3 cells. (b) CCK-8 assay was performed to determine whether RAPGEF4 overexpression could rescue the reduced growth capacity caused by NOP2 knockdown. (c-d) cell migration and invasion assays were performed to determine whether RAPGEF4 overexpression could rescue the reduced migration and invasion ability caused by NOP2 knockdown. (e) RNA m^5^C dot blot assay of RNA m^5^C methylation levels in NOP2 knockdown and overexpressed Hey and Caov3 cells, methylene blue staining (as control). (f) m^5^C-RIP and qRT-PCR assays were performed to analyze the m^5^C methylation levels of RAPGEF4 mRNA in NOP2 knockdown Hey and Caov3 cells and the corresponding wild-type cells. (f) Co-IP assay revealed the interaction of RAPGEF4 with NOP2 in NOP2 and RAPGEF4 overexpressed Hey cells. *: *p* < .05. **: *p* < .01 ***: *p* < .001.

Extraction of cytoplasmic and nuclear lysates and Western Blot assay showed that NOP2 was distributed in both the nucleus and cytoplasm of HGSOC cells, with a predominant expression in the nucleus (Figure 5a). Subsequently, we simultaneously transfected the NOP2 knockdown plasmid and RAPGEF4 overexpression plasmid and found that the elevated expression of RAPGEF4 rescued the decrease in cell proliferation, migration, and invasion caused by reduced NOP2 expression as determined by CCK-8, cell migration, and cell invasion assays (Figure 5b-d). RNA m^5^C dot blotting assay showed that decreasing NOP2 expression reduced m^5^C levels, while increasing NOP2 expression raised m^5^C levels (Figure 5e). Through m^5^C-RIP and qRT-PCR assays, we found that the knockdown of NOP2 decreased the m^5^C methylation level of RAPGEF4 mRNA (Figure 5f). We also found evidence of an interaction between NOP2 and RAPGEF4 through CO-IP assay (Figure 5g).

## Discussion

Ovarian cancer is a prevalent gynecologic tumor and a leading cause of cancer-related deaths in women globally. HGSOC is the most common histologic subtype of ovarian cancer, accounting for 70–80% of ovarian cancer deaths, and is associated with poor prognosis and frequent recurrence. Despite the availability of sophisticated treatments, ovarian cancer patients have unsatisfactory 5-year survival rates because of late diagnosis and limited understanding of ovarian cancer pathogenesis, which limits available treatment strategies. Therefore, the exploration of the mechanisms of HGSOC and the identification of new diagnostic markers are critical.

RNA m^5^C modification is a crucial post-transcriptional modification of RNA. Recent reports have identified m^5^C-associated methyltransferases, with NSUN2 being the most prevalent in tumors. NSUN2 has been shown to promote the proliferation of gastric cancer cells by inhibiting cyclin-dependent kinase inhibitor 1C (p57^Kip2^) or interacting with small ubiquitin-like modifier (SUMO)-2/3,^39,40^ and has been implicated in breast, cervical, gallbladder, esophageal squamous cell, and uveal melanoma cancers,^41–45^ as well as being related to worse prognosis in pancreatic cancer and squamous carcinoma of the head and neck.^46,47^ However, NOP2 has not been extensively studied in tumors. NOP2 has been described to enhance the cell proliferation, migration and invasive ability of colon cancer,^18^ and is associated with poor prognosis in renal clear cell carcinoma, gastric adenocarcinoma and ovarian cancer.^17,48,49^ To date, mechanistic studies of NOP2 in ovarian cancer have not yet been reported. In this study, we found that the protein level of NOP2 was higher in HGSOC than in FT. Subsequent experiments revealed that NOP2 significantly enhanced HGSOC cell proliferation, migration, and invasion *in vitro*, and tumor growth *in vivo*.

We subsequently identified the cAMP signaling pathway as a major pathway regulated by NOP2 in HGSOC based on RNA-seq and KEGG analysis. The cAMP signaling pathway has a broad role in cells. EPAC proteins, including two isoforms Epac1 and Epac2, are one of its major downstream targets. Epac2 is also known as RAPGEF4. Epac proteins are involved in several biological processes, including gene transcription, cell proliferation and apoptosis, and tumorigenesis and development. RAPGEF4 can affect the sensitivity of lung cancer cells to cisplatin by altering cAMP levels through the Epac2-Rap1A-Akt pathway^50^ and is relevant to the pathogenesis of glioma.^51^ Although RAPGEF4 is not the only target of NOP2, our experiments demonstrated that RAPGEF4 is one of the downstream genes regulated by NOP2. In our study, we found that the expression levels of NOP2 and RAPGEF4 were positively correlated, and the expression of RAPGEF4 was decreased in the NOP2 knockdown cell lines. And there is a direct or indirect relationship between NOP2 and RAPGEF4. Our results provided a possible regulatory mechanism of NOP2 and RAPGEF4 in HGSOC.

According to previous studies, RNA methyltransferases have been demonstrated to have a major function in mRNA transcription, translation and nuclear export, particularly in tumors.^52^ NSUN2 is currently the most extensively studied RNA methyltransferase and is primarily associated with RNA stability. The functional mechanism of NOP2 has been poorly studied to date, with current research mainly focused on nucleolus generation and cell proliferation.^15,53^ In our experiments, we found that NOP2 was involved in the proliferation, migration, and invasion of HGSOC. NOP2 is predominantly found in the nucleus, but is also present in the cytoplasm. Current studies of NOP2 have focused on its role in ribosome synthesis and processing, however, our findings suggest that there may be another way in which NOP2 acts in HGSOC that can influence the progression of HGSOC. In NOP2 knockdown cells, both total RNA level and mRNA m^5^C methylation level of RAPGEF4 is reduced. The elevated expression of RAPGEF4 rescued the decrease in cell proliferation, migration, and invasion caused by reduced NOP2 expression. Further experiments revealed a direct or indirect relationship between NOP2 and RAPGEF4. Based on these results, we propose a regulatory mechanism of NOP2 on RAPGEF4 that is dependent on the m^5^C methylation level.

## Conclusion

In this study, we explored the effect of NOP2 on the cAMP signaling pathway in HGSOC cells and found an effect of NOP2 expression on the prognosis and proliferation of HGSOC. Eventually, we demonstrated that there might be a regulatory mechanism between NOP2 and RAPGEF4 dependent on m^5^C methylation levels.

## Supplementary Material

Supplemental Material

## Data Availability

The GEO database in this study can be found online. The raw data of the RNA-Seq used in the study have been uploaded to NBCI. Submission ID is: SUB13324231.

## Abbreviations

HGSOC: High-grade serous ovarian cancer
FT: Fallopian tubes
FTE: Fallopian tube epithelium
m^5^C: 5-Methylcytosine
m^6^A: N6-methyladenosine
m^1^A: N1-methyladenosine
m^7^G: 7-methylguanosine
NOP2: nucleolar protein p120
NSUN1: NOP2/Sun RNA Methyltransferase Family Member 1
NSUN2: NOP2/Sun RNA Methyltransferase Family Member 2
Writers: Methyltransferases
Erasers: Demethyltransferases
Readers: Associated binding proteins
FPKM: Fragments per kilobase of exon model per million mapped fragments
TCGA: The Cancer Genome Atlas
GEO: Gene Expression Omnibus
CNV: Copy number variation
OV: Ovarian Cancer
OS: Overall Survival
DEGs: Differentially Expressed Genes
GO: Gene Ontology
KEGG: Kyoto Encyclopedia of Genes and Genomes
CO-IP: Co-immunoprecipitation
GSEA: Gene set enrichment analysis
RNA-seq: RNA sequencing
cAMP: Cyclic adenosine monophosphate
PKA: Protein kinase A
Epac: Exchange protein directly activated by cAMP
GEFs: Guanine nucleotide exchange factors
RAPGEF4: Rap guanine nucleotide exchange factor 4
IOD: Integrated Optical Density
AOD: Average Optical Density
OD: Optical Density
SUMO: small ubiquitin-like modifier
p57Kip2: Cyclin-dependent kinase inhibitor 1C
