# Supplementary figures and images for: Function of m5C RNA methyltransferase NOP2 in high-grade serous ovarian cancer
Source: Cancer Biol Ther. 2023 Oct 6;24(1):2263921. doi: 10.1080/15384047.2023.2263921 (PMC10561575; doi:10.1080/15384047.2023.2263921)

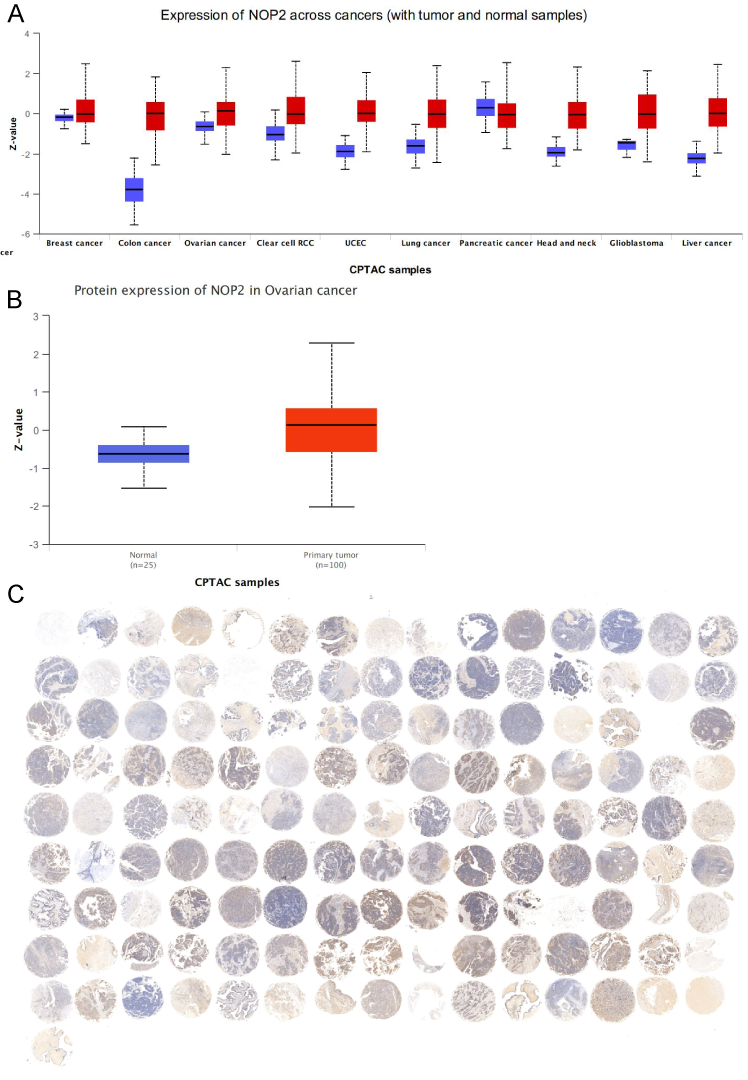

Supplement: Supplemental Material [file KCBT_A_2263921_SM3080.zip › Figure S1.tif]

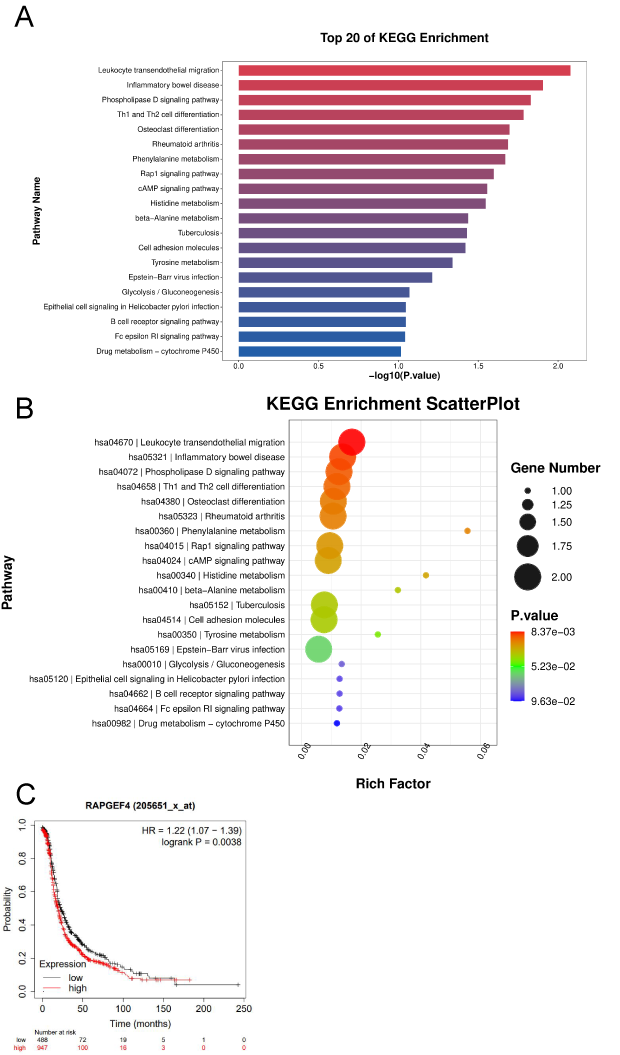

Supplement: Supplemental Material [file KCBT_A_2263921_SM3080.zip › Figure S2.tif]
