# Supplementary material for: Function of m5C RNA methyltransferase NOP2 in high-grade serous ovarian cancer
Source: Cancer Biol Ther. 2023 Oct 6;24(1):2263921. doi: 10.1080/15384047.2023.2263921 (PMC10561575; doi:10.1080/15384047.2023.2263921)
Supplement: Supplemental Material [file KCBT_A_2263921_SM3080.zip › Supplementary Legends.doc]

**Supplementary Legends**

**Figure S1.** Supplementary Figure S1: (A) Box plot showing protein expression of NOP2 in 10 types of cancer in CPTAC database. (B) Box plot showing protein expression of NOP2 in ovarian cancer in CPTAC database. (C) NOP2 immunohistochemistry analysis of tissue chip consisting of 27 normal ovarian (13 FT tissues) and 93 ovarian cancer (63 HGSOC tissues) paraffin sections.

**Figure S2.** Supplementary Figure S2: (A) The first 20 KEGG-enriched pathways. (B) Scatter of the KEGG enrichment pathway. (C) Wound healing assay of NOP2 overexpressing Hey cells. (D) Kaplan–Meier OS analysis of RAPGEF4 expression in OV patients.
